# Nursing students’ understanding of caring for older people receiving home care: a qualitative study

**DOI:** 10.1186/s12877-026-07975-w

**Published:** 2026-07-11

**Authors:** Merita Neziraj, Elisabeth Carlson, Malin Sundström, Maria Samuelsson

**Affiliations:** 1 Department of Care Science, Malmö University, Jan Waldenströms gata 25, Malmö, 205 06 Sweden; 2 Kristianstad University, Kristianstad, Sweden

**Keywords:** Education, Home care, Nursing, Students, Older people

## Abstract

**Background:**

Global demographic ageing trends and changes in healthcare require that nursing students are adequately prepared to care for older people in home care settings. However, many students report insufficient knowledge and feel unprepared for this role. The aim of this study was to describe the variation in nursing students’ understanding of caring for older people receiving home care. This study forms part of a broader project aiming at developing a tailored educational intervention in collaboration with nursing students to prepare them for this role.

**Methods:**

A qualitative study with a phenomenographic approach was conducted. Eighteen individual semi structured interviews were conducted with nursing students at three Swedish universities between February and April 2025. The interviews were conducted either via telephone, digitally via Zoom or face-to-face and subsequently analysed.

**Results:**

The results comprise three descriptive categories illustrating the variation in nursing students’ understanding of caring for older people: *Nurses’ collaborative role in tailoring person-centred care for older people receiving home care*,* Challenges to person-centred care for older people receiving home care* and *Opportunities to person-centred care for older people in the home care setting.* Within these understandings, students emphasised that nurses need to collaborate with older people, their family members and colleagues to tailor person-centred care to older people at home. However, students also identified challenges that could hinder person-centred care for older people, inducing factors related to older people, nurses and the organisation. Despite these challenges, students recognised opportunities inherent in the home care setting, such as the potential to develop deeper relationships with older people, support their family members, and engage with the broader community.

**Conclusions:**

The results indicate that students recognised the essential collaborative role nurses play in tailored person-centred for older people in this setting, while also identifying challenges that may hinder its full realisation. At the same time, the students pointed to concrete opportunities for strengthening person-centred approaches within home care. These insights deepen our understanding of how nursing students conceptualise care for older people receiving home care and provide insight into how an educational intervention can be developed to better equip them to meet the complex needs of older people in this setting.

**Supplementary Information:**

The online version contains supplementary material available at 10.1186/s12877-026-07975-w.

## Background

The global growth of the older population represents one of humanity’s greatest achievements, reflecting advances in medicine, health and overall living conditions [1]. Increased longevity is often regarded as a marker of societal progress, yet this demographic change also introduces profound challenges for health care systems worldwide. Accompanied by the growing number of older people, one of the most pressing areas in health care today is the transformation of care provided in home care settings. In this study, home care refers to professional healthcare provided at home to older people with formally assessed needs, including rehabilitative, supportive, and technical nursing care [2].

Nursing students, as future health care providers, play a pivotal role in addressing the transformation of home care. As a part of the larger project Prepare to Prevent (described further in the methods section), the present study describes nursing students’ understanding of caring for older people receiving home care. Hence, the study not only contributes to a deeper understanding of how future nurses conceptualise care for older people in this setting but also provides insight into how an educational intervention can be developed to prepare them for their role.

Globally, the proportion of older people is expected to increase dramatically. The proportion of those aged 60 years and above will nearly double from 12% to 22% between 2015 and 2050, reaching approximately 2.1 billion people [3]. The most substantial growth, however, will occur among those aged 80 and older, whose number will triple by 2050, amounting to approximately 423 million [3]. In parallel, demographic projections for Sweden indicate that by 2030, at least 30% of the population will be aged 65 years and above, while the number of those aged 80 years and above is expected to increase from 530,000 to 800,000 [4]. This represents a positive demographic trend and today’s older population is generally more active, independent and healthier than in previous generations [5]. Additionally, the traditional view of the passive patient has been replaced by a more person-centred perspective, which recognises older people as active participants in their own care [6]. Furthermore, older people often express a strong preference to remain in their own homes as they age [7], and in many countries, including Sweden, this preference is supported by ageing-in-place policies that emphasise enabling older people to remain in their homes for as long as possible.

Nonetheless, ageing among older people can be associated with increased negative health outcomes [8]. Nearly half of older people receiving home care are considered frail [9] and have severe comorbidities and cognitive dysfunction [8]. Moreover, among older Swedish persons living at home and receiving home care, approximately 48% are at risk of developing malnutrition and 30% poor oral health. Additionally, 14% are at risk of developing pressure ulcers, while 64% face a heightened risk of falling [10].

Registered nurses (hereafter referred to as nurses) play a fundamental role in providing home care [11]. However, nurses often report lacking the theoretical knowledge and practical skills needed to provide high-quality care in this setting [12]. Moreover, owing to the absence of nurses in the clinical learning environment, nursing students are sometimes supervised by nursing assistants, which may imply inadequate clinical supervision and support according to a systematic review [13]. In addition to these challenges, it has been reported that nursing students have expressed negative attitudes towards home care in general [14], especially home care for older people [15]. Moreover, nursing students have inadequate knowledge about caring for older people [16]. This might explain why they often express feelings of unpreparedness for their future roles in home care [17], suggesting an urgent need for educational intervention to meet their needs in this regard. Nonetheless, to our knowledge there are currently no educational interventions specifically designed to address the needs of nursing students in caring for older people in this setting. This gap underscores the need for improved education along with clinical training. Consequently, the results from the current study provide an essential foundation for the development of future educational interventions aimed at strengthening nursing students’ knowledge and preparedness within the home care setting.

### Aim

The aim of this study was to describe the variation in nursing students’ understanding of caring for older people receiving home care.

## Methods

The current study is a part of the larger research project Prepare to Prevent. Following the Medical Research Council (MRC) framework for developing, evaluating and implementing complex interventions [18], Prepare to Prevent is designed to develop, evaluate and implement an educational intervention aimed at preparing nursing students to provide care for older people receiving home care, as well as for involving and supporting their family members. This study aligns with the first phase of the MRC farmwork, i.e., the development phase, and focuses specifically on nursing students’ understanding of caring for older people receiving home care.

### Design

The study was designed as a qualitative study with a phenomenographic approach. This approach strives to describe the variation in the ways a group understands a phenomenon rather than the range of understanding for each individual within the group [19]. Furthermore, phenomenography describes how a phenomenon is conceived (a second-order perspective), not how something is in fact (a first-order perspective [19].

### Setting

Swedish universities offer a three-year bachelor’s nursing program, comprising 180 European Credits Transfer System credits. The program contains equal parts theory and clinical practice, resulting in not only a bachelor’s degree but also a licence to practice as a registered nurse. In addition to national qualification objectives, there are local qualification objectives. Additionally, nursing programs can have different educational structures, for example, when students practice in home care during the program.

### Recruitment and participants

Three universities in southern Sweden were invited to participate in the study. The department heads at these universities agreed to participate and approved the study. The inclusion criteria required students in their first and final years to be enrolled at one of these universities, with or without clinical experience in home care. Using the phenomenographic approach [19], students were purposefully recruited to capture the diverse ways in which the phenomenon of caring for older people receiving home care is understood, irrespective of their previous experiences with the topic students.

At each university, lecturers who were not involved in the study disseminated written information about the study through their respective learning platforms. Additionally, they identified suitable opportunities to verbally inform students about the study. On these occasions, which took place either digitally or face-to-face, they provided a brief verbal introduction to the study, followed by more detailed information presented by members of the research team (MN, MSa, and EC). Students were also given a QR code for easy access to study details and to share their contact information if they were interested in participating. Those who expressed interest were then contacted by MN (the first author) and MSa (the last author), who provided a comprehensive information letter and an opportunity to ask questions, as well as to arrange the format and timing for the interview. A total of 18 students participated in the study. For participants’ characteristics, see Table 1.

**Table 1:** Characteristics of participants (*n* = 18) regarding gender, age, university and semester

Characteristics of the participants	
Gender, n (female/male)	16/2
Age, mean (range)	33 (22–47)
university, n (A/B/C)	6/1/11
Semester, n (semester 1/5/6)	14/3/1

### Data collection

Data were collected through individual semistructured interviews between February and April 2025. MN and MSa conducted the interviews following an interview guide. To test and calibrate the interview guide and the recording equipment, two pilot interviews were conducted with students, followed by a discussion between MN and MSa. As no amendments to the interview guide were deemed necessary, the pilot interviews were included in the dataset.

Before the interviews started, the participants were reminded of the study’s aim and procedures and that there were no right or wrong answers to help them feel at ease. They were also informed that the interviews could last as long as they wished, ensuring that they could stop at any time without any consequences. The interviews started with the collection of background information, including information about the students’ enrolled university, sex and age. The interviews then followed with a broad opening question: *When I say caring for older people receiving home care*,* what are your thoughts?* Participants were also asked probing questions such as *Can you explain a bit further?* to encourage deeper reflection to capture the second-order perspective.

The interviews were conducted either via telephone (*n* = 11), digitally via Zoom (*n* = 5) or face-to-face (*n* = 2), all in private rooms. With the participants’ permission, 17 of the interviews were audio recorded. One of the students preferred not to be audio recorded; hence, this interview was documented in writing. During data collection, MN and MSa continually discussed the data and evaluated them for redundancy, concluding the process after 18 interviews when no new variation in students’ understanding was identified. The interviews lasted between 30 and 57 min, with a median duration of 45 min.

### Data analysis

With the participants’ permission, the interviews were transcribed verbatim, and these transcripts constituted the dataset for analysis. During the analysis, we followed the seven-step iterative process outlined by Sjöström and Dahlgren [20]. First, to *familiarise* us with the dataset, MN and MSa listened to all the interviews and thoroughly read the transcripts, correcting any errors encountered. Additionally, MN reread the transcripts to ensure a deep understanding of the dataset. During the *compilation step*, MN identified and highlighted elements that were pertinent to the study’s aim and made marginal notes. These elements were then documented in a Word document by MN and shared with the research team. During the *condensation step*, these elements were reduced to their core meanings. During the *grouping step*, similar elements were grouped into descriptive categories. Then, during the *articulating step*, the groups were contrasted and compared to establish clear boundaries between the categories. In this phase, the research team moved iteratively back and forth between the third and fifth steps, discussing the categories and revising the preliminary categories until three descriptive categories were generate and *named* to capture their essence. During the *contrastive comparison step*, the categories were further refined to clearly capture the distinction between each category. In this process, attention was given not to only to clarifying the distinctive understanding of each category, but also how they related to one another. This involved identifying similarities across the categories and describing their structural relationships. Through this process, the hierarchy and the logic relations among categories became visible, presented in an *outcome space* [19]. Consistent with phenomenographic approach, the outcome space presented in the results section reflects a structured set of different ways of understanding the phenomenon, in which each category is distinctive, nonoverlapping, and in relation to each other [19].

### Rigor

Throughout the study design, data collection, analysis, and reporting, the trustworthiness criteria outlined by Lincoln and Guba [21] and the criteria for a credible phenomenographic study [22] were considered. To minimise involuntary influence on the process and the description of the nursing students’ understanding, the preconceptions of the authors involved in data collection and analysis were discussed and documented. This was followed up regularly, and the preliminary results were reviewed in light of these early notes. For credibility, the interviews followed a piloted interview guide, were audio recorded, and were transcribed verbatim. Moreover, the researchers who conducted the interviews had experience in qualitative interviewing and the research field. The analysis was conducted iteratively and in collaboration between the researchers, who all had experience in conducting qualitative analysis. Furthermore, quotations are integrated into the results. To enhance dependability, methodological choices were documented and reported using the COREQ checklist [23], and the interview guide is accessible (Supplemental files). To enhance transferability, the characteristics of the participants are presented.

### Ethical considerations

The study underwent ethical review and was deemed not to fall under the requirements of the Swedish Ethical Act for empirical studies and therefore was granted only an advisory opinion (Dnr 2024-05260-01). Nevertheless, the study adhered to the principles of the Declaration of Helsinki [24]. To ensure informed participation, all participants were thoroughly informed about the study’s aim and procedures and were given sufficient time to consider participation. Informed consent was obtained before participation, and the interviews were scheduled at participants’ convenience. They were also assured that they could withdraw from the study at any time without any consequences. The research team consists of lecturers at two of the universities; however, they were not involved in teaching or grading the courses during the recruitment and data collection phases to ensure a balance of power.

## Results

The analytic process of describing variation in the students’ understanding of caring for older people receiving home care generated three descriptive categories: *Nurses’ collaborative role in tailoring person-centred care for older people receiving home care*,* Challenges to person-centred care for older people receiving home care* and *Opportunities to person-centred care for older people in the home care setting.*

### Nurses’ collaborative role in tailoring person-centred care for older people receiving home care

This category comprises three subcategories and describes students’ understanding that tailoring person-centred care requires nurses to collaborate with the older people, their family members, and colleagues.

#### Tailoring care through collaboration with older people

The students believed that collaboration with older people is shaped by each person’s diverse life experiences and personal preferences, highlighting that no two older people are the same. Within this understanding, nurses were seen as needing to attend to older people’s overall life and home situation to tailor care to meet their specific needs. For instance, some students noted that while certain older people enjoy spending time outdoors, others prefer the comfort of staying at home, illustrating how personal preferences can influence how care could be tailored. Further, students described that nurses should actively seek to consider each person’s preferences and incorporate these into the care process, allowing care to be guided by older persons themselves. Students believed that such collaboration could support more meaningful care. Further, students stressed that nurses should always strive to provide the highest level of care possible, based on the best interests of older people, regardless of the circumstances.

*“Yes*,* every time I am in contact with at patient (older person)*,* I try to*,* or rather*,* I always ask them (older people) how they want things to be…then I ask how they would like things to be. We try to work in a person-centred way*,* and that is why I at least try to stay in continues contact with the patients (older people). We talk about these things together”.* (Participant 15)

The students also highlighted that collaboration with older people should be shaped by their individual personality traits. Older persons were described by some students as open-minded and sociable, while others were described as more introverted or even cynical. Within this variation, students emphasized the importance of nurses to adapting their communication style. For instance, if an older person appreciates humour, nurses should be attentive this and adjust their tone, whereas a more serious demeanour may be appropriate for others. Regardless of these differences, students emphasised that nurses should maintain a respectful and responsive attitude, tailoring their approach to match each older person’s unique character when delivering care in their own homes. This was considered especially important when identifying individual solutions to address older people’s care needs to achieve positive health outcomes.

*“You still have people around you who are understanding*,* who listen*,* and who show respect*,* that these (older people) are individuals who have lived full lives and carry a wealth of experience and knowledge*,* and that they are given the respect that older people deserve”.* (Participant 2)

### Tailoring care through collaboration with family members

They students highlighted the inclusion of older people’s family members, recognising them as valuable collaborative partners within this setting. The students believed that most older people prefer to have their family members involved in their care; as such, nurses should actively collaborate with their family members since their involvement could enhance both the quality and effectiveness of care. For instance, family members often have deeper insight into older people’s preferences, making their inclusion valuable throughout tailoring care. However, some students also acknowledged that not all older people have strong relationships with their family members. Therefore, they highlighted the importance of nurses’ professional judgement in determining when and how family members can be appropriately involved in care.

*“They (older people) have*,* you know*,* a family relationship or some other close relationship. So*,* in one sense*,* you’re (as a nurse) a bit of an outsider*,* but you also have a relationship with the patient – just a completely different kind of relationship. It can be difficult to balance that and to know how much I can say*,* how much am I allowed to say*,* how much should I say?”* (Participant 11)

#### Tailoring care through collaboration with colleagues

The students regarded collegial collaboration within the home care setting as decisive in enabling nurses to deliver tailored care. Within this perspective, collaboration with different professionals, such as physicians, physiotherapists, and occupational therapists, was emphasised as essential. Students expressed that, although nurses are required to draw on broad and comprehensive knowledge in order to identify symptoms and determine appropriate, individualised care interventions within the home environment, effective care is not only shaped through collaboration with fellow nurses. Rather, it also involves close engagement with nursing assistants, who often spend more time with older people and therefore may have deeper insight into their care needs and progression over time, thereby supporting the delivery of tailored care. At the same time, while some students took for granted the availability of collegial support, others raised concerns about situations in which nurses may work in isolation, without access to colleagues for consultation. For these students, teamwork was therefore viewed as extending beyond a mere practical arrangement.

“*Collaboration, for example with nurse assistants, could involve some way of signalling to them when something has been missed, so that they can in turn notify their registered nurse*”. (Participant 2)

### Challenges to person-centred care for older people receiving home care

This category comprises three subcategories and describes students’ understanding that person-centred care for older people receiving home care involves several challenges. These challenges were related to the older people, the nurses and the organisation.

#### Challenges related to the older people

The students identified several challenges associated with older people that may hinder person-centred care in a home care setting. These challenges were described as spanning physical, emotional, environmental, and cognitive dimensions. For instance, although many older people at home may wish to engage in their own care, some students believed that physical limitations could hinder their ability to do so, making it difficult for them to follow nurses’ individualised care recommendations. Other students thought that emotional challenges may be linked to older people’s unwillingness to replace familiar items, such as their regular beds with hospital-style beds, which may hinder nurses to deliver care to their specific care needs. Additionally, the presence of nursing equipment in the home was described as potentially undermining the comfort and familiarity of the older people’s living environment, while also making nurses’ work more difficult to meet their care needs. Some students also noted cognitive challenges that might arise, particularly when older people have dementia, leading to a lack of insight into their situation and to noncompliance with care recommendations, which could worsen their health.

*“And also*,* that older people don’t always understand - not just because of language*,* but because it feels like there’s another communication barrier*,* where they sometimes don’t take in information as well.”* (Participant 10)

#### Challenges related to the nurses

The students believed that challenges to person-centred care for older people receiving home care could be related to nurses’ personalities. Nurses’ personal characteristics were described as potentially influence the quality of care. For instance, a domineering or overly authoritative character was described as less suited to the home care setting, as this could negatively affect individualised care.

*“That’s were a conflict can arise*,* because in this perspective she (the nurse) could have a more overarching viewpoint*,* almost a kind of dominance technique…When you are dependent (older)*,* especially if you have multiple conditions*,* you rely in others. And if someone (nurses) has a narrow or limited perspective*,* it can be devastating”.* (Participant 2)

Further, the students feared that a lack of comprehensive knowledge and limited clinical experience could hinder nurses from delivering care effectively. To bridge this gap, the students proposed a thorough course covering the ageing process of older people, including preventive efforts to support healthy ageing in place. Furthermore, establishing an on-campus clinical training centre designed to mirror the home environment would provide valuable hands-on experiences in caring for older people and better prepare them for future clinical practice as well as work in the field.

*“So*,* I think there should be more focus on lifting techniques and so on. How to care for older people in their homes. In my opinion*,* that should be focused more. It easily becomes very hospital-focused in the clinical training centre*,* since it is more like a hospital ward”.* (Participant 4)

#### Challenges related to the organization

On the organisational level, the challenges to person-centred care were more closely linked to the nature of the care environment, which could impact the patient safety of older people, the nurses’ work environment, and the nurses’ personal safety.

The students believed that caring for older people receiving home care could be problematic in several ways. For instance, they noted that the dual nature of the environment: on one hand, it was the older people’s private home and personal space, while on the other hand, it served as the nurse’s workplace. This dynamic introduced logistic limitations. The students raised concerns about hygiene-related difficulties that could risk patent safety, particularly in areas such as wound care. Some students raised additional concerns included long travel distances and time constraints, which could further impede individual care. They also noted safety risks for nurses, such as the inability to contact colleagues because of technical problems in suburban areas, thereby placing themselves in potentially dangerous situations.

*“ It can be pretty tricky*,* I think… Like*,* where do you put things? And how do you work? I find it tough working in someone’s home.”* (Participant 13)

### Opportunities to person-centred care for older people in the home care setting

This category comprises three subcategories and describes students’ understanding that the home care setting offers valuable opportunities for person-centred care, such as deepening relationships with older people, facilitating support to their family members, and involving the broader community.

#### The home care setting offers opportunities to deepen relationships with older people

The students highlighted that the home care setting, in contrast to other settings, offers nurses the possibility to engage in comprehensive introductory conversations with older people. These encounters were seen as enabling nurses to assess older people’s resources and strengths in a careful and holistic manner, thereby prevent further illness and promote health.

*“You will get to know the patients*,* establish and try to build a good relationship to understand their needs…That’s something I find very valuable*,* when you can have a longer period care. For example*,* in home care*,* you meet the same people repeatedly*,* so that you can build relationships. You can develop relationship with the nurses as a patient”.* (Participant 18)

Further, some students described how the home care setting facilitate regular encounters with older people, which in turn supports getting to know them better and developing strong bonds. Within this understanding, motivating older people was considered important in helping them recognise the value and benefits of a preventive and health-promoting approach. In addition, students emphasised that nurses should fully utilise the home care environment to deepen their engagement, for instance, by connecting with older people through old photographs or personal interests to make care more meaningful and personalised.

#### The home care setting facilitates supporting the older people’s family members

The students described the home care setting as offering opportunities for nurses to establish relationships not only with older people but also with the family members. The home care setting was suggested as unique possibility to consider the health and well-being of family members themselves. From this perspective, the home care setting was understood as enabling nurses to be attentive to the needs of family members’ aside older people.

*“They think it’s difficult having their mother at home. Yes*,* but why do they find it difficult? Do they feel that they have the main responsibility when they actually don’t need to carry that responsibility. I mean*,* we should involve the family members as well*,* that’s what it’s about. Because family caregiving is very important”. *(Participant 5)

#### The home care setting offers opportunities to involve the broader community

The students described the involvement of the broader community as a further opportunity within the home care, suggesting such engagement could support and promote healthy ageing. Other students viewed the municipality as having an important role in facilitating activities for older people in the community. Furthermore, involving residential associates was seen as a valuable resource. Such community engagement could, in turn, contribute to enhanced well-being among older people.

*“ So it doesn’t have to be that difficult. These things are already out there. Lots of places - mostly churches and similar - have cafés*,* soup lunches*,* and all those activities that you know they tend to go to.”* (Participant 7)

#### Outcome space

The descriptive categories *Challenges to person-centred care for older people receiving home care* and *Opportunities to person-centred care for older people in the home care setting* are distinct from to each other yet essential to the category *Nurses’ collaborative role in tailoring person-centred care for older people receiving home care.* This category serves as the foundation for the others, as challenges can impede, whereas opportunities can strengthen nurses’ collaborative role in tailoring person-centred care for older people in this setting. In turn, all categories are related to the importance of ensuring person-centred care for older people receiving home care.

## Discussion

The present study aimed to describe the variation in nursing students’ understanding of caring for older people receiving home care. The results show that the students emphasize the importance of ensuring person-centred care for older people receiving home care. To ensure person-centred care, students believed that nurses need to collaborate with older people, their family members and colleagues in order to tailor care to older people in this setting. However, person-centred care was understood to be challenged by factors related to the older people themselves, nurses, and the organisation. Despite these challenges, the students identified opportunities to deepen relationships with older people, being able to support their family members and involve the community to strengthen person-centred care within the home care setting. In the following section, nurses’ collaborative role in tailoring care for older people in home care, along with the related challenges and opportunities, will be discussed in relation to person-centred care.

In line with the person-centred nursing framework [6], our results indicate that students recognised the importance of nurses collaborating with older people in order to tailor care. Specifically, students expressed that older people’s life situations, experiences and personal preferences need to be taken into account in care provision. This could be understood in the light of the need to truly know older people, which is essential for supporting older people’s autonomy and their crucial role in shared decision making in line with person-centre care [6], thereby strengthening older people’s sense of involvement in their own care. At the same time, a scoping review indicates that this emphasis on respecting autonomy and preferences may, at times, conflict with ensuring care that aligns with patient safety [25]. This potential tension highlights the complexity of balancing person-centred care, as nurses must navigate situation in which older people’s wishes may not always correspond with recommended care. Consequently, this underscores the importance of addressing such ethical and practical tensions within nursing education.

The students viewed person-centred care as challenging rather than straightforward. Several challenges were related to the older people themselves. Students noted, for instance, that health conditions could limit older people’ ability to follow care recommendations. They also explicitly raised concerns about how cognitive difficulties may hinder older people’ adherence to care recommendations. This is not surprising given that many older people living at home experience cognitive impairments [9]. Students’ concerns are highly relevant in the Swedish context, and internationally, up to 150,000 people are living with dementia, the majority of whom remain in their own homes [26]. Furthermore, Lethin and colleagues [27] found that persons with dementia who live at home have more severe behavioural and psychological symptoms than those living in nursing homes, which further complicates the provision of holistic nursing care as outlined in the person-centred care framework [6]. This highlights the need to integrate comprehensive theoretical knowledge about dementia into nursing education, particularly in the home care context.

Challenges were also related to the nurses as individuals. The students expressed concerns that certain personality traits among nurses could negatively influence the provision of person-centred care. It is well established that personality traits may influence our thoughts and behaviour, and some researchers even suggest that personality may influence the choice of nursing specialty [28]. Importantly, this underscores the need to consider students’ personalities during their education to support them in clinical training and ensure that they can achieve learning outcomes [29]. Moreover, students emphasised the importance of possessing comprehensive knowledge to accurately identify symptoms and determine appropriate interventions that reflect the preferences and values of older people. However, they expressed concerns that may relate to gaps in nurses’ knowledge, suggesting that the necessary prerequisites for truly person-centred care are difficult to fully meet. Within the person-centred nursing framework, prerequisites such as professional competence and well-developed interpersonal skills are emphasised as critical for nurses [6]. The results of our study therefore point to a gap between the theoretical expectations of person-centred care and the practical realities encountered by students, underscoring the need for enhanced education and clinical training to strengthen both expertise and skills in home care settings. Previous research has shown that as students progress in their studies, they demonstrate increasingly positive attitudes towards and knowledge about older people [15], suggesting that one single course may be insufficient to meet their needs. Instead, as suggested by the students in our study, person-centred care for older people in home care could be integrated as a continuous thread through nursing education, ensuring that students consistently develop competence and confidence in caring for this population.

Despite these challenges, the students believed that the home care setting offered meaningful opportunities for nurses to provide person-centred care. This is consistent with the person-centred care framework [6], in which the care environment is conceptualised as a core component shaping the extent to which person-centred care can be provided. According to the students, this specific setting, enabled nurses to build strong relationships with older people, thereby strengthening the provision of person-centred care. Such relationships were suggested to be deepen trough longer conversations and by paying closer attention to older people’s personal preferences, which highlights the importance of working with the person’s believes and values to improve good care experience [6]. Importantly, a previous study conducted in the home care setting with older people showed that older people value deeper conversations with healthcare professionals, as these play a crucial role in providing conformation and understanding [30]. Moreover, supporting family members was recognised as a valuable opportunity in the home care setting. However, although students identified opportunities to support family members in this setting, and despite the person-centred nursing framework clearly emphasising the importance of involving family members to achieve holistic nursing care [6], previous research indicates that their support needs are unmet in setting [31, 32]. This highlights the importance of addressing the role and needs of family members more explicitly in nursing education.

### Limitations

The inclusion of nursing students from three universities enabled us to capture the broad variation in students’ understanding of caring for older people receiving home care. Given that previous research has reported negative attitudes among students towards home care for older people [15], it is possible that students in our study provided desirable answers, which may have influenced the results. To mitigate this risk, we made efforts to create a safe and open environment and informed students prior to the interviews that there were no wrong or right answers. Another limitation might be that the results reflect a national and context-specific perspective. Nevertheless, we have provided demographic information about the participants and the setting to support transferability. Moreover, the demographic shift towards an ageing population is a global phenomenon, suggesting that the results of the current study may be relevant to similar contexts internationally. Another limitation concerns the use of telephone interviews. While this data collection method has been criticised for reducing the richness of qualitative interviews [33], it also offers important advantages. Conducting interviews by telephone enabled participation from students across different universities. This flexibility was particularly valuable for engaging students with varying schedules and clinical practices.

## Conclusions

This study represents the first phase of a larger project aimed at developing an educational intervention to better prepare nursing students for the demographic shift towards an older population receiving home care. The students in the study recognised the essential collaborative role that nurses play in providing tailored person-centred care to older people at home, yet they also identified challenges that may hinder the realisation of such care in practice. Moreover, they highlighted meaningful opportunities for nurses to enhance the provision of person-centred care within this setting. Importantly, the results have several educational implications. The students emphasised the need for nursing education to more explicitly address the complexities of caring for older people in their homes. They highlighted the importance of covering a breadth and depth of theoretical knowledge about ageing in general, dementia, and family involvement *and* integrating this knowledge into a home-like training environment on campuses that mirrors the reality in practice. Incorporating these areas within the curriculum may better equip future nurses to provide high-quality, person-centred care to an increasingly older population receiving home care. Taking the results of the current study into consideration, the next step is to conduct further research aimed at developing a tailored educational intervention that better prepares nursing students for their role in caring for older people receiving home care.

## Supplementary Information

Supplementary Material 1.

Supplementary Material 2.

## Data Availability

The datasets generated and analysed during the current study are not publicly available due to the nature of the research and ethical considerations.

## Abbreviations

MRC: The Medical Research Council framework for developing, evaluating and implementing complex interventions
COREQ: The COnsolidated criteria for REporting Qualitative research) Checklist

## References

[1] World Health Organisation. Ageing: Global population. Available from: Ageing: Global population. https://www.who.int/news-room/questions-and-answers/item/population-ageing.

[2] Genet N, Boerma WG, Kringos DS, Bouman A, Francke AL, Fagerström C, et al. Home care in Europe: a systematic literature review. BMC Health Serv Res. 2011;11:207. 21878111 10.1186/1472-6963-11-207 PMC3170599

[3] World Health Organisation. Ageing and Health. Available from: Ageing and health. https://www.who.int/news-room/fact-sheets/detail/ageing-and-health.

[4] Neziraj M, Axelsson M, Kumlien C, Hellman P, Andersson M. The STAIR OF KNOWLEDGE—a codesigned intervention to prevent pressure ulcers, malnutrition, poor oral health and falls among older persons in nursing homes in Sweden: development of a complex intervention. BMJ Open. 2023;13(8):e072453. 37562934 10.1136/bmjopen-2023-072453 PMC10423781

[5] Falk H, Johansson L, Ostling S, Thøgersen Agerholm K, Staun M, Høst Dørfinger L, et al. Functional disability and ability 75-year-olds: a comparison of two Swedish cohorts born 30 years apart. Age Ageing. 2014;43(5):636–41. 24595067 10.1093/ageing/afu018

[6] McCance T, McCormack B. The Person-centred Nursing Framework: a mid-range theory for nursing practice. J Res Nurs. 2025;30(1):47–60. 40093819 10.1177/17449871241281428 PMC11907491

[7] Bonifas RP, Simons K, Biel B, Kramer C. Aging and place in long-term care settings: influences on social relationships. J Aging Health. 2014;26(8):1320–39. 25502244 10.1177/0898264314535632

[8] Tasioudi L, Aravantinou-Karlatou A, Karavasileiadou S, Almegewly WH, Androulakis E, Kleisiaris C. The Impact of Frailty and Geriatric Syndromes on the Quality of Life of Older Adults Receiving Home-Based Healthcare: A Cross-Sectional Survey. Healthcare. 2023;11(1):82. 10.3390/healthcare11010082 PMC9819361 36611542

[9] Stratidaki E, Mechili EA, Ouzouni C, Patelarou AE, Savvakis I, Giakoumidakis K et al. An Investigation of the Risk Factors Related to Frailty in Older Adults Receiving Home Care Services. Nutrients. 2024;16(23). 10.3390/nu16233982. 10.3390/nu16233982 PMC11643836 39683377

[10] Neziraj M, Hellman P, Kumlien C, Andersson M, Axelsson M. Prevalence of risk for pressure ulcers, malnutrition, poor oral health and falls - a register study among older persons receiving municipal health care in southern Sweden. BMC Geriatr. 2021;21(1):1–10. 33882869 10.1186/s12877-021-02205-x PMC8059027

[11] Ranta L, Kaunonen M. Requirements for nurses’ competencies in home care: An integrative review – a mixed-method approach. Nordic J Nurs Res. 2024;44:20571585241270286.

[12] Silverglow A, Johansson L, Lidén E, Wijk H. Perceptions of providing safe care for frail older people at home: A qualitative study based on focus group interviews with home care staff. Scand J Caring Sci. 2022;36(3):852–62. 34423863 10.1111/scs.13027

[13] Oshodi TO, Sookhoo D. Nursing students’ perceptions of inadequate nurse staffing in the clinical learning environment – a systematic narrative review. Nurse Educ Pract. 2025;82:104221. 39647175 10.1016/j.nepr.2024.104221

[14] Watson J, Horseman Z, Fawcett T, Hockley J, Rhynas S. Care home nursing: Co-creating curricular content with student nurses. Nurse Educ Today. 2020;84:104233. 31731223 10.1016/j.nedt.2019.104233

[15] Castro C, Antunes R, Simoes A, Bernardes C, Fernandes JB. Nursing students’ knowledge and attitudes toward older adults. Front Public Health. 2023;11:1150261. 37900038 10.3389/fpubh.2023.1150261 PMC10600372

[16] Baumbusch J, Dahlke S, Phinney A. Nursing students’ knowledge and beliefs about care of older adults in a shifting context of nursing education. J Adv Nurs. 2012;68(11):2550–8. 22364192 10.1111/j.1365-2648.2012.05958.x

[17] Donmez E, Dolu i, Kurklu. Nursing students’ perspectives on the quality of nursing care in-home health care. Med Sci. 2021;10(2):321–7.

[18] Skivington K, Matthews L, Simpson SA, Craig P, Baird J, Blazeby JM, et al. A new framework for developing and evaluating complex interventions: update of Medical Research Council guidance. BMJ-BRITISH Med J. 2021;374:n2061. 10.1136/bmj.n2061 PMC8482308 34593508

[19] Marton F. Phenomenography - Describing conceptions of the world around us. Instr Sci. 1981;10:177–200.

[20] Sjöström B, Dahlgren LO. Applying phenomenography in nursing research. J Adv Nurs. 2002;40(3):339–45. 12383185 10.1046/j.1365-2648.2002.02375.x

[21] Lincoln YS, Guba EG. Naturalistic inquiry: sage. 1985.

[22] Stenfors-Hayes T, Hult H, Dahlgren MA. A phenomenographic approach to research in medical education. Med Educ. 2013;47(3):261–70. 23398012 10.1111/medu.12101

[23] Tong A, Sainsbury P, Craig J. Consolidated criteria for reporting qualitative research (COREQ): a 32-item checklist for interviews and focus groups. Int J Qual Health Care. 2007;19(6):349–57. 17872937 10.1093/intqhc/mzm042

[24] World Medical Association. Declaration of helsinki ethical principles for medical research involving human subjects. 2013 https://www.wma.net/policies-post/wma-declaration-of-helsinki-ethical-principles-for-medical-research-involving-human-subjects/. 10.1001/jama.2013.281053 24141714

[25] Perone AK, Zhou L, Glusker A, Shin J, Xu C, Thairani P et al. A Scoping Review on Strategies for Navigating Conflicting Rights Between Safety and Autonomy in Residential Long-Term Care in the United States. Journal of the American Geriatrics Society.n/a(n/a). 10.1111/jgs.70532 PMC13496346 42302162

[26] Neziraj M, Striberger R, Samuelsson M. Prevention of pressure ulcers, malnutrition, poor oral health, and falls among older persons in nursing homes in Sweden - healthcare professionals’ experiences of participating in a co-design project: A qualitative study. Geriatr Nurs. 2025;64:103433. 40602001 10.1016/j.gerinurse.2025.103433

[27] Lethin C, Rahm Hallberg I, Vingare E-L, Giertz L. Persons with Dementia Living at Home or in Nursing Homes in Nine Swedish Urban or Rural Municipalities. Healthcare. 2019;7(2):80. 31242681 10.3390/healthcare7020080 PMC6627377

[28] Kennedy B, Curtis K, Waters D. Is there a relationship between personality and choice of nursing specialty: an integrative literature review. BMC Nurs. 2014;13(1):40. 25516719 10.1186/s12912-014-0040-z PMC4267136

[29] Axelsson M, Jakobsson J, Carlson E. Which nursing students are more ready for interprofessional learning? A cross-sectional study. Nurse Educ Today. 2019;79:117–23. 31125764 10.1016/j.nedt.2019.05.019

[30] Axén A, Christiansen L, Taube E, Kumlien C, Borg C. The Significance of Social Connections and Health in Relation to Loneliness Experienced by Older Adults Living at Home: A Qualitative Study. Scand J Caring Sci. 2025;39(2):e70057. 40551561 10.1111/scs.70057 PMC12186019

[31] Oosterveld-Vlug MG, Custers B, Hofstede J, Donker GA, Rijken PM, Korevaar JC, et al. What are essential elements of high-quality palliative care at home? An interview study among patients and relatives faced with advanced cancer. BMC Palliat Care. 2019;18(1):96. 31694715 10.1186/s12904-019-0485-7 PMC6836458

[32] Midlöv EM, Sterner T, Porter S, Lindberg T, Forss KS. Standing next to but not being part of: relatives’ experiences of support from healthcare professionals when general palliative care is provided at home. BMC Palliat Care. 2026;25(1). 10.1186/s12904-026-02021-3 PMC12930732 41689015

[33] Ward K, Gott M, Hoare K. Participants’ views of telephone interviews within a grounded theory study. J Adv Nurs. 2015;71(12):2775–85. 26256835 10.1111/jan.12748

