# Supplementary material for: Nursing students’ understanding of caring for older people receiving home care: a qualitative study
Source: BMC Geriatr. 2026 Jul 11;26:955. doi: 10.1186/s12877-026-07975-w (PMC13371020; doi:10.1186/s12877-026-07975-w)
Supplement: Supplementary file 2 — Supplementary Material 2. [file 12877_2026_7975_MOESM2_ESM.docx]

**Interview guide**

The aim of this study was to describe the variation in nursing students’ understanding of caring for older people receiving home care

| *Opening question: When I say care for older people receiving home care – what are your thoughts?* | |
| --- | --- |
| *Specific questions about older people receiving home care*   - Prevention of illness - Promote health - The role of a registered nurse   *Specific questions about older people receiving home care in nursing education*   - Nursing education - Learning - Gaps - Preferences | *Follow up questions:*   - Hinders and possibilities? - Why do you think that is? - Could you give an example? - What do you mean with…? - What was good/less good? - Why? - Intervention desing? - Intervention format? - Intervention delivery? |
| *Closing question: Is there anything that I have missed or that you would like to add?* | |
